# Leukemia’s Next Top Model? Syngeneic Models to Advance Adoptive Cellular Therapy

**DOI:** 10.3389/fimmu.2022.867103

**Published:** 2022-03-25

**Authors:** Jaquelyn T. Zoine, Sarah E. Moore, M. Paulina Velasquez

**Affiliations:** ^1^ Department of Bone Marrow Transplantation and Cellular Therapy, St. Jude Children’s Research Hospital, Memphis, TN, United States; ^2^ Graduate School of Biomedical Sciences, St. Jude Children’s Research Hospital, Memphis, TN, United States

**Keywords:** adoptive cell immunotherapy, leukemia, syngeneic animal model, leukemia microenvironment, cell therapy

## Abstract

In recent years, there has been an emphasis on harnessing the immune system for therapeutic interventions. Adoptive cell therapies (ACT) have emerged as an effective option for B-cell derived hematological malignancies. Despite remarkable successes with ACT, immune dysregulation and the leukemia microenvironment can critically alter clinical responses. Therefore, preclinical modeling can contribute to the advancement of ACT for leukemias. Human xenografts, the current mainstay of ACT *in vivo* models, cannot evaluate the impact of the immunosuppressive leukemia microenvironment on adoptively transferred cells. Syngeneic mouse models utilize murine tumor models and implant them into immunocompetent mice. This provides an alternative model, reducing the need for complicated breeding strategies while maintaining a matched immune system, stromal compartment, and leukemia burden. Syngeneic models that evaluate ACT have analyzed the complexity of cytotoxic T lymphocytes, T cell receptor transgenics, and chimeric antigen receptors. This review examines the immunosuppressive features of the leukemia microenvironment, discusses how preclinical modeling helps predict ACT associated toxicities and dysfunction, and explores publications that have employed syngeneic modeling in ACT studies for the improvement of therapy for leukemias.

## Introduction

Adoptive cell therapy (ACT) is the expansion and infusion of immune cells, including natural killer (NK) cells, gamma-delta (γδ) T cells, and alpha-beta (αβ) T cells, into patients for therapeutic benefit. Advancements in the field of ACT have resulted in engineered cellular products that express performance-enhancing receptors, such as cytokine receptors, T cell receptors (TCRs), or chimeric antigen receptors (CARs). Overall, the innovation of genetically engineered immune cells in the ACT setting has resulted in improved outcomes, especially for patients with B-cell derived hematologic malignancies (1, 2). However, challenges remain like antigen selection and overcoming an immunosuppressive microenvironment (3–5).

Despite promising preclinical ACT data, patients can fail to respond to treatment once a strategy is translated into the clinic. One of the many factors contributing to failed ACT is a highly immunosuppressive tumor microenvironment (TME), causing adoptively transferred cells to become dysfunctional or exhausted (6). Structural components, soluble factors, and immune cells found within the leukemic TME contribute to a hostile environment in the bone marrow niche, which poses a threat to adoptively transferred cells. Leukemic blasts can reprogram both the structural components of the microenvironment and the function of immune cell populations, allowing for a more favorable environment for leukemia progression (7).

These immune interactions are understudied in preclinical ACT models. The most frequently used preclinical model to determine ACT anti-leukemia activity is a xenograft model. Xenograft models assess human cellular products against human cells, making it feasible to study multiple human-derived cell lines with different genetic drivers of leukemogenesis. However, xenografts lack a functional immune system and tumor heterogeneity found in leukemia patients preventing them from having the necessary rigor to predict clinical responses (8, 9). Therefore, there is room for improvement in preclinical modeling to achieve continued development of effective immunotherapies for leukemias.

Syngeneic models encompass allografts of mouse tumors in immunocompetent mice. This allows for evaluation of toxicities, including on-target/off-tumor side effects, and the immunosuppressive microenvironments (8). Immunocompetent models have not been readily adapted to ACT studies, in part, because of the difficulty to isolate and expand murine immune cells, lack of homology between targeted proteins, and cross-reactivity of CAR T cells’ single chain variable fragments (scFv). Despite inherent hurdles to establishing syngeneic models, they provide an avenue to ensure the optimization of ACT against hematological malignancies. In this review, we evaluate the role of the bone marrow niche and leukemia microenvironment on leukemogenesis, assess available models to test ACT, and discuss literature that utilizes syngeneic modeling to evaluate ACT.

## Bone Marrow Niche/Leukemia Microenvironment

**Figure 1:**
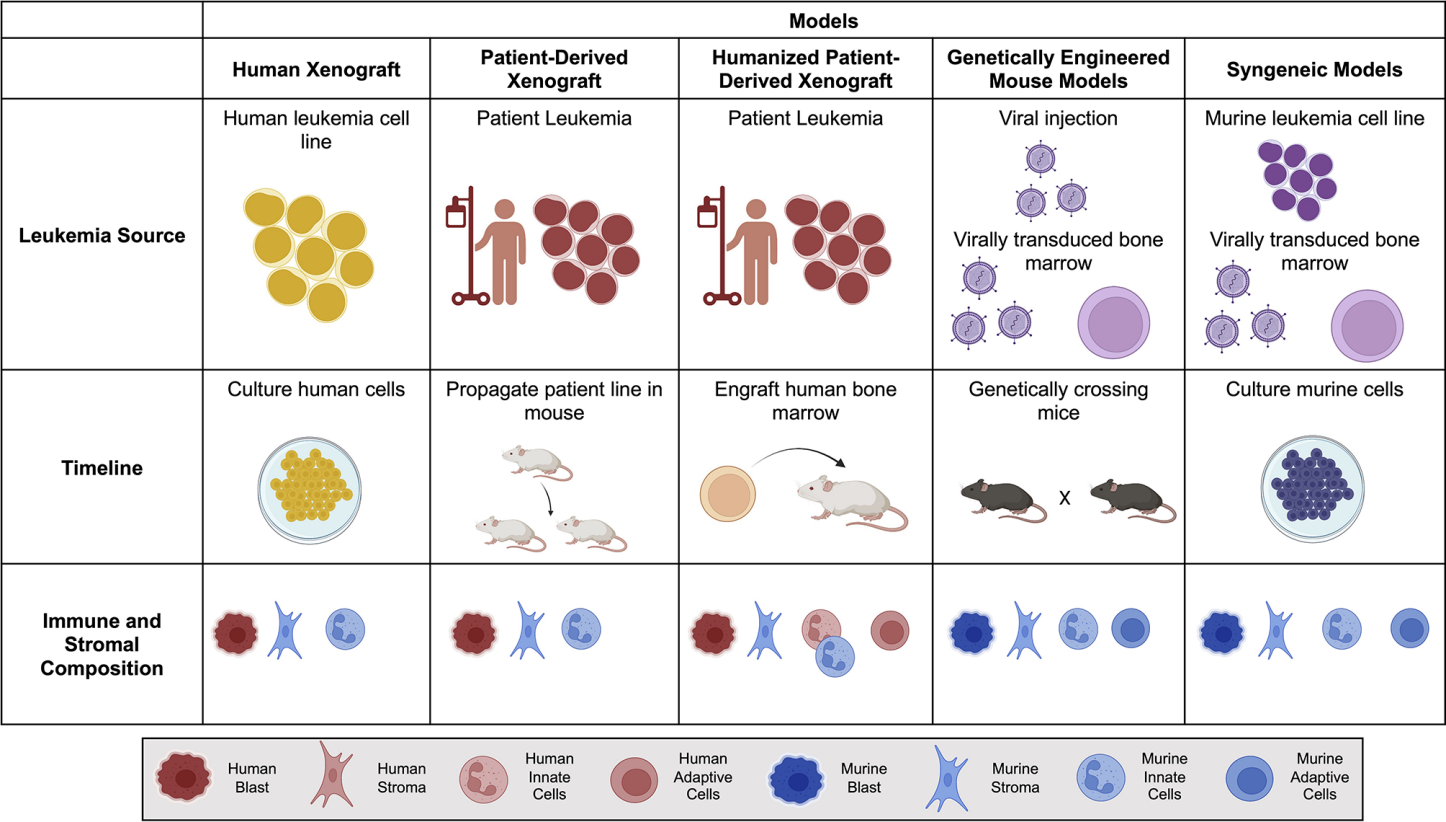
Comparison of mouse models for Adoptive Cell Therapy (ACT). Brief overview of the leukemia source, timeline to propagate and test tumor model, and immune and stromal composition of each mouse model. Human is denoted by warm colors (yellow, orange, red) and murine is denoted by cool colors (blue and purple). Created with BioRender.com.

The bone marrow microenvironment is essential for the pathogenesis and progression of leukemias (10, 11). The niche provides a physical sanctuary site for developing rare populations of leukemic cells and harbors an immunosuppressive environment that downregulates the natural immune surveillance required to eliminate tumor cells successfully. When choosing a model of ACT, it is essential to consider structural and immune components within each *in vivo* model (**Figure 1**). As investigators design new cellular immunotherapies, there has been an emphasis on understanding the impact the TME will have on therapeutic success.

There are two major patterns in leukemia relapse: i) the initial clone gains mutations; or ii) a subclone survives initial treatments (12). In most cases the relapse clone is characterized as a leukemia stem cell (LSC) (13). LSCs have distinct properties from bulk leukemic cells, such as limitless self-renewal and initiation of leukemia. LSCs are inherently less susceptible to traditional chemotherapeutics and can escape immune surveillance (14).

Structurally, the bone marrow contains two anatomically different hematopoietic stem cell (HSC) niches, known as the central and endosteal, that are crucial for the production and maintenance of healthy HSCs (15, 16). Within these compartments, HSCs are regulated by endothelial, osteoblastic, and stromal cell components, specifically mesenchymal stromal cells (MSCs) (17, 18). MSCs are multipotent cells that make up most of the structural components of the bone marrow stroma. In a leukemic state, MSCs play a large role in the leukemia pathogenesis through two major mechanisms: 1) providing physical protection of leukemic cells and 2) reprogramming of bone marrow niche (14, 19). Thus, MSCs and the bone marrow stroma play an important role in leukemia progression and relapse but are not commonly considered in most models of ACT.

Soluble factors, such as cytokines, chemokines, and enzymes, are important components of the TME that suppress the endogenous immune response and support leukemia progression. Compared to the healthy bone marrow landscape, leukemia cytokine signatures show an increase in transforming growth factor β (TGF-β) and hepatocyte growth factor (HGF) levels (20). These two factors help mediate T cell suppression and reduce expression of NK cells (20, 21). Increases in anti-inflammatory cytokine, interleukin (IL)-10, are observed in a variety of leukemia models, and often limit ACT functionality (22). Chemokines play an important role in both trafficking of leukemic cells and cellular immunotherapies. The CXCL12-CXCR4 chemokine pathway, is involved in the homing of HSCs within the bone marrow (23). CXCL12 secreted by the bone marrow along with the upregulated expression of CXCR4 on leukemia blasts increases the homing of tumor cells to the bone marrow (24). Once the leukemic blasts are within the bone marrow niche, they are protected structurally, capable of secreting anti-inflammatory soluble factors, and dysregulating immune cell populations.

In addition, concentrations of certain enzymes within the bone marrow contribute to leukemia progression (23, 24). Blasts mediate expression of arginase II, promoting a low arginine microenvironment. The limited arginine drives monocytes to a suppressive phenotype while suppressing T cell expansion (5). Indoleamine 2,3-dioxygnenase (IDO) is also released by blasts, which converts CD4+ T cells into T regulatory cells (Tregs), thus enhancing the suppressive capacity of the microenvironment (5).

Leukemia-induced remodeling of the TME alters the structural and chemical components within the bone marrow and influences immune cell populations. The leukemia microenvironment comprises innate (dendritic cells [DC] and macrophages) and adaptive (myeloid derived suppressor cells [MDSCs], Tregs, and NK cells) immune cells. Leukemic cells hinder the maturation of DCs, often promoting immune tolerance and thus inducing the development of Tregs (25). TME-associated macrophages can be inhibitory or stimulatory, but their inhibitory function diminishes the anti-tumor activity of adoptively transferred cells within the TME (26). MDSCs arise from myeloid progenitors and are a subset of immature myeloid cells that lead to NK-cell dysfunction and recruitment of Tregs, among other immunosuppressive cells (27, 28). They are difficult to model and contribute to the failure of many AML therapeutic interventions, making them a potential therapeutic target.

The bone marrow microenvironment plays an aggressive role in leukemia progression, highlighting the importance of using preclinical models to evaluate interactions between the host immune system, leukemic TME, and adoptively transferred cells. However, there is limited work analyzing the contribution of the immune system and microenvironment on effective cellular therapies for leukemia. The advancement of these interventions relies on the active exploration and adaptation of preclinical modeling, and especially in the syngeneic context.

## Preclinical Leukemia Modeling for ACT

### Xenograft Models

The initial development of ACTs for leukemias has been aided by using human xenografts. These models have allowed functional evaluation of human cell therapy products against human cell lines or patient tumors (patient-derived xenograft- PDX). In addition, they have facilitated high throughput screening of many ACT interventions. PDXs provide a heterogenous leukemia model but lack a comprehensive and intact immune system required to adequately study ACT interventions. In addition, human T cells can recognize mouse xenoantigens in this setting, increasing the risk of graft-versus-host disease. Alternatives to human xenografts include using humanized PDXs, genetically engineered mouse models (GEMMs), and syngeneic mouse models (8, 9).

### Humanized Models

PDXs are the best option to increase the heterogeneity of leukemic burden. The major difference between a humanized or non-humanized PDX model is the reconstitution of human immune cells in the immunocompromised mouse (29). Immune reconstitution is not maintained for long periods due to the high turnover of bone marrow cells and decreased engraftment of human cells within a mouse (29, 30). There is no guarantee that each immune population will reconstitute within the mouse, leading to differences in the humanized immune system and an inaccurate or incomplete representation of an immune system (30). In humanized models, there is an added challenge of patient leukemia cells engrafting within the same timeframe of complete immune cell reconstitution. Humanized PDXs are time-consuming to establish with a low yield of implantation and are not reliable when screening multiple interventions in a timely manner.

### Genetically Engineered Mouse Models

GEMMS are unique murine models which genetically manipulate the somatic activation of oncogenes or inactivation of tumor suppressors to elicit *de novo* tumor development (31). Leukemias that arise from these genetic alterations typically mimic histological and molecular features of human disease. An advantage to this model is the maturation of leukemia cells within an immunocompetent host. This allows researchers to analyze scenarios of immune pressure on leukemia development which can then result in genomic instability. While this strengthens the tumor heterogeneity within the system, murine leukemia can mature to express unique tumor-associated antigens between mice with the same genetic manipulation (9). This makes studies utilizing GEMMs challenging to reproduce because of their genetic drift within “equivalent” models. Because ACT, such as CAR T cells, rely on targeting a tumor antigen on the surface of leukemia cells, GEMMs do not provide the consistency to measure antigen specificity. Also, without knowing the surface proteins on each mouse in a GEMM experiment it is difficult to study immune escape mechanisms, such as lineage switch or antigen down-regulation (9). Additionally, these systems can be unpredictable with variable latencies and penetrance.

### Syngeneic Mouse Models

Syngeneic models use murine cell lines or virally-transduced murine HSCs to express genes of interest (i.e., oncogene amplification, knock out tumor suppressors, overexpress fusion proteins) that result in leukemia initiation (32). They do not require the complex breeding necessary in GEMMs but do not have the advantage of leukemic development within the native immune system. They offer a rigorous option to test cellular immunotherapies due to their rapid growth, reproducibility, intact immune system, and hostile leukemia microenvironment. However, they can lack heterogeneity and there are few readily available leukemia options (9).

To bypass the lack of immune system in human leukemia models, the complicated breeding of GEMMs, and the difficulty in generating relevant syngeneic leukemia tumor models, researchers have expressed human antigens on readily available murine ALL tumor cells to test CAR T cells (8, 9, 32). However, this is confounded by the potential of the mouse’s endogenous immune response to recognize the human antigen, making it difficult to discern the cause of the autoimmune response (8, 9, 32). It is noteworthy that GEMMs and syngeneic models utilize mouse biology to draw conclusions on human therapeutic interventions. In addition, they are difficult to adapt to replicate cell-based immunotherapy for hematologic malignancies successfully. Despite this, given the significant contributions the immune system and TME have on leukemia progression and relapse, it is essential to accurately mimic these systems for successful ACT evaluation.

## Syngeneic Models Evaluating ACT for Heme Malignancies

There are limited syngeneic leukemia models that have been used for ACT evaluation. While several murine B-ALL models exist, the C1498 cell line has served as one of the only commercially available murine AML cell lines and has been sparingly used in ACT research (33–35). The studies presented below highlight their utility, allowing for toxicity evaluation as well as the careful mechanistic dissection of ACT.

### Syngeneic Models Evaluating Cytotoxic T-Lymphocyte (CTL) or Transgenic TCR Responses

As early as 1981, investigators used *in vitro* sensitization or immunization to generate murine lymphocytes specific to murine tumor antigens. Cheever et al. isolated splenocytes from BALB/c mice and cultured them *in vitro* in the presence of virally-induced syngeneic leukemia (LSTRA), an ascitic lymphoma originally induced in newborn BALB/c mice with Moloney leukemia virus. This exposure was meant to sensitize the murine lymphocytes to LSTRA (36). They did not find any direct evidence supporting a link between *in vitro* culture of lymphoid cells with LSTRA and increased antitumor activity *in vivo.* However, they observed that depletion of the T cell population diminished antitumor effectors, demonstrating the importance of T cells in antitumor activity (36). Subsequently, another syngeneic leukemia model expanded tumor-specific T cells from spleens of FBL-3 (friend virus-induced erythroleukemia) *ex vivo*. Although they determined that immunized mice responded to antigen *in vivo*, they also observed that antigen naive T cells extracted from mice became dependent on IL-2, limiting the therapeutic potential of the T cells. They overcame this hurdle by exposing the extracted T cells to anti-CD3 antibody and IL-2 (37). These studies were instrumental in the advancement of tumor-infiltrating lymphocyte (TIL) therapy tumor models.

Several syngeneic systems have been used to assess leukemia-specific immune responses. Mumprecht et al., for example evaluated responses to 2 different CML models including a chronic (BCR/ABL) and a blast crisis CML (BCR/ABL-NUP98/HOXA9) model that had been previously described (38, 39). Mumprecht et al. determined that mice that received a lower tumor burden and had disease elimination developed a LCMV-gp33- specific CTL response, while mice that had CML progression lacked persistence of CTLs (40). In a follow up study, they determined that elimination of CD8+ T cells in a CML model led to disease progression and that IL-7 secreted by CML helped maintain a CTL response, leading to stable disease as it is characteristic of chronic phase CML (41). This data highlights the importance of syngeneic modeling cellular immunotherapies to pursue effective non-cellular therapy-based combinations.

Zhou et al. used C57BL/6 bearing C1498 murine AML to evaluate the impact of Tregs on adoptively transferred tumor-reactive CTLs (35). They showed that anti-AML reactive CTLs had potent antitumor activity *in vitro* but not *in vivo*, due to the presence of tumor-localized Tregs. To bypass this hurdle, they pretreated tumor bearing mice with IL-2 diphtheria toxin restoring CTL proliferation and effect.

Syngeneic leukemia models have also been utilized to evaluate responses and the biology of transgenic TCRs, highlighting their versatility to investigate the effects of ACT. One group of investigators evaluated long-lasting antitumor activity of CD8+ T cells specific to the gag epitope of an oncogenic Friend murine leukemia virus (FMuLV) model (42, 43) and confirmed leukemia control after injection of T cells expressing a transgenic TCR. Other investigators have used a syngeneic C1498 model to better understand mechanisms of immune evasion using TCR transgenic mice (34).

### Syngeneic Models for CAR T Cell-Based Immunotherapy

CD19-CAR T cells have improved outcomes for patients with relapsed/refractory B cell malignancies. However, xenograft mouse models used to test the CD19-CAR are limited in determining how T cell function is affected by Tregs, possible off-target/on-tumor activity of the CAR, and possible immune rejection of adoptively transferred T cells. With that in mind, Cheadle et al. designed a first generation murine CD19-CAR (mCD19-CAR) which allowed for temporary tumor regression in an A20 murine lymphoma model. Importantly, mCD19-CAR infusion did not result in any overt toxicities (44). Kochenderfer et al. subsequently generated a second generation CAR that achieved reduction in lymphoma burden, albeit with limited CAR T cell persistence (45). This corresponds with comparisons between first- and second-generation CAR constructs in humans and reiterates the importance of a costimulatory domain for enhanced antitumor activity.

Davila et al. subsequently tested this mCD19-CAR in a Eμ-ALL01 B-ALL model, a leukemia with similar genetic and cellular characteristics as adult human B-ALL (46). In this study, they were able to prove that mCD19-CAR T cells recognize and kill Eμ-ALL01 leukemia cells. They also noted that CD8+ mCD19-CAR T cells allowed for long-term tumor control. Most importantly, the established syngeneic model allowed them to dissect the effects of lymphodepletion and T cell dose on the effector function of CAR T cells.

In addition, B-ALL models have been used to further investigate complications stemming from ACT. One group used a E2aPBX murine pre-B ALL model to study the function granule-mediated cytotoxicity in anti-mCD19-CAR T cell efficacy (47). Researchers knocked out perforin from mCD19-CAR T cells and discovered perforin was not required for cytotoxicity and when tested *in vivo*, perforin knockout CD19-CAR T cells produced more proinflammatory cytokines than WT counterparts (47). This led to the mice developing hemophagocytic lymphohistiocytosis (HLH)-like toxicities.

Furthermore, Jacoby et al. demonstrated lineage switch after mCD19-CAR T cell therapy, evaluating late relapses in 2 different B-ALL models (E2a.PBX and Eμ-RET) (4). They demonstrated that Eμ-RET leukemia did not show lineage switch upon relapse after mCD19-CAR T cell treatment. However, mice bearing E2a:PBX exposed to mCD19CAR T cells underwent lineage switch upon relapse, showing downregulation of Pax5 and Ebf1. They could recapitulate this lineage switch by deletion of Pax5 or Ebf1. This study further demonstrated the utility of syngeneic models in the quest to optimize CAR T cell therapy for hematological malignancies.

## Conclusion

Developing effective ACT for leukemias still poses several challenges requiring a better understanding of both the adoptively infused cells and the TME. Although clinical trials provide the ultimate test for ACT, murine models can be a powerful tool to gain insight. One of the largest drawbacks of current preclinical modeling of leukemia targeted ACTs, is that it heavily relies on xenografts which lack a representative immune system and TME. Syngeneic models offer an alternative to better evaluate these factors. However, the availability of certain leukemia syngeneic models, such as AML, are still limited and establishing new systems can often be time consuming and unreliable (8, 9, 32). Additionally, it is not always possible to adapt human-target ACT towards respective murine antigen counterparts. For example, the evaluation of CAR T cell therapies is limited by finding an antigen recognition domain (i.e. scFv) that recognizes the corresponding cell surface murine antigen. In addition, trafficking of adoptively transferred cells to the TME can greatly affect the efficacy of treatment (48). Thus, several factors that impact homing, such as target antigen expression, immune cell populations (25–28, 48), and chemokine production (23) are important to recognize and incorporate into preclinical modeling. Syngeneic models provide these factors and allow for a better understanding of immune cell trafficking to the tumor site. Nevertheless, the field of syngeneic experimentation has adapted to include additional genetic modifications on cellular products such as cytokine receptors on mCAR T cells (49). The analysis of ACT therapies in syngeneic models can aid in answering critical questions and warrants further exploration and development.

## Author Contributions

JZ, SM, and MV conceptualized the manuscript and provided content. All authors reviewed, edited, and approved the final manuscript.

## Funding

This work was supported by the Assisi Foundation of Memphis and the American Lebanese Syrian Associated Charities (ALSAC).

## Conflict of Interest

MV and JZ hold patent applications in the field of gene and cell therapy.

The remaining author declares that the research was conducted in the absence of any commercial or financial relationships that could be construed as a potential conflict of interest.

## Publisher’s Note

All claims expressed in this article are solely those of the authors and do not necessarily represent those of their affiliated organizations, or those of the publisher, the editors and the reviewers. Any product that may be evaluated in this article, or claim that may be made by its manufacturer, is not guaranteed or endorsed by the publisher.

## Abbreviations

ALL, Acute lymphocytic leukemia; AML, Acute myeloid leukemia;ACT, Adoptive cell therapy; αβ, Alpha-beta; CARs, Chimeric antigen receptors; CLL, Chronic lymphocytic leukemia; CML, Chronic myeloid leukemia; CTL, Cytotoxic T-lymphocyte; DC, Dendritic cell; FMuLV, Friend murine leukemia virus; FBL-3, Friend virus-induced erythroleukemia; γδ, Gamma-delta; GEMM, Genetically engineered mouse model; HSC, Hematopoietic stem cell; HLH, Hemophagocytic lymphohistiocytosis; HGF, Hepatocyte growth factor; IDO, Indoleamine 2,3-dioxygenase; IL, Interleukin; LSC, Leukemia stem cell; MSCs, Mesenchymal stromal cells; mCD19-CAR, Murine CD19-CAR; MDSC, Myeloid derived suppressor cells; NK, Natural killer; PDX, Patient derived xenograft; scFv, Single chain variable fragment; TCRs, T cell receptors; Tregs, T regulatory cells; TGF- β, Transforming growth factor β; TIL, Tumor-infiltrating lymphocyte; TME, Tumor microenvironment; LSTRA, Virally-induced syngeneic leukemia.
